# Potenziale der Bakteriophagentherapie in Deutschland: Evidenzlage und klinische Relevanz

**DOI:** 10.1007/s00103-025-04048-y

**Published:** 2025-04-28

**Authors:** Felix Bröcker, Christian Willy

**Affiliations:** 1 Idorsia (Berlin) Pharmaceuticals GmbH, Magnusstr. 11, 12489 Berlin, Deutschland; 2 Klinik für Unfallchirurgie und Orthopädie, Septisch-Rekonstruktive Chirurgie, Forschungs- und Behandlungszentrum Rekonstruktion von Defektwunden, Bundeswehrkrankenhaus Berlin, Scharnhorststraße 13, 10115 Berlin, Deutschland

**Keywords:** Phagentherapie, Antibiotikaresistenz, Multiantibiotikaresistenz, Einzelfallbehandlung, Klinische Studien, Phage therapy, Antibiotic resistance, Multidrug resistance, Case studies, Clinical trials

## Abstract

Die Bakteriophagentherapie (kurz: Phagentherapie) zeigt großes Potenzial, den Problemen der uns bedrohenden zunehmenden Infektionen mit Multiantibiotikaresistenzen entgegenzuwirken. Registerstudien und systematische Übersichtsarbeiten zeigen, dass Phagenbehandlungen für diverse Indikationen bei etwa 80–90 % der antibiotikaresistenten Infektionen wirksam sind. Die jährlich deutschlandweit etwa 62.000 Fälle von Infektionen durch resistente oder schwer behandelbare Erreger könnten prinzipiell mit Phagentherapien behandelt werden. Momentan laufen mehrere klinische Studien zur Sicherheit und Wirksamkeit der Phagentherapie für spezifische Indikationen, es hat jedoch noch kein Präparat ein Zulassungsverfahren durchlaufen. Zudem wird die zeitnahe Anwendbarkeit der Phagentherapie für Einzelfallbehandlungen durch hohe Produktionskosten, begrenzte Kapazitäten und regulatorische Hürden eingeschränkt. Erste Marktzulassungen in den USA und Europa werden in den nächsten 3–5 Jahren erwartet, was die Perspektiven der Phagentherapie erheblich stärken könnte. Konkrete Schritte zur Beschleunigung der Anwendung der Phagentherapie in Deutschland zur Entlastung des Gesundheitssystems wie die Einrichtung eines Phagenherstellungsbetriebs, der Abbau regulatorischer Hürden für Einzelfallbehandlungen, die Förderung innovativer Technologien für Herstellung und Diagnostik und die Förderung klinischer Zulassungsstudien werden genannt.

## Abstract (en)

Bacteriophage therapy (phage therapy) holds great potential in addressing the growing threat of infections caused by multidrug-resistant bacteria. Registry studies and systematic reviews indicate that phage treatments are effective in approximately 80–90% of antibiotic-resistant infections across various indications. In principle, the approximately 62,000 annual cases of infections caused by resistant or difficult-to-treat pathogens in Germany could be treated with phage therapy. Currently, several clinical trials are underway to assess the safety and efficacy of phage therapy for specific indications; however, no product has yet undergone a formal approval process. Furthermore, the timely applicability of phage therapy for individual cases is constrained by high production costs, limited capacities, and regulatory hurdles. The first market approvals in the USA and Europe are expected within the next three to five years, which could significantly strengthen the outlook for phage therapy. Concrete steps to accelerate the implementation of phage therapy in Germany to relieve the healthcare system include establishing a dedicated phage manufacturing facility, reducing regulatory barriers for compassionate use, promoting innovative technologies for production and diagnostics, and supporting clinical approval studies.

## Einleitung

Die Belastung des Gesundheitssystems durch Infektionen mit Erregern mit Vielfachresistenzen gegen Antibiotika (multiresistente Erreger – MRE) ist in Deutschland erheblich (siehe Artikel „Gesundheitsökonomische Bedeutung antimikrobieller Resistenzen“ in diesem Themenheft). Vor diesem Hintergrund erlebt die Phagentherapie als Alternative bzw. zusätzliche Behandlungsmöglichkeit zu Antibiotika momentan eine Renaissance, da mit Phagen auch antibiotikaresistente Bakterien bekämpft werden können [1].

Erste Erfolge wurden bereits vor 100 Jahren berichtet. Felix d’Hérelle behandelte 1919 in Paris erfolgreich Kinder mit schwerer Ruhr mit Phagen [2]. Ab den 1930er-Jahren produzierten Firmen wie Behring (Deutschland) und Eli Lilly (USA) Phagenpräparate.

Die Einführung von Antibiotika in den 1940ern, unsachgemäßer Phagengebrauch, Unklarheiten über deren Natur (Elektronenmikroskope waren erst ab den späten 1930er-Jahren verfügbar) und Herstellungsprobleme führten zum Niedergang der Phagentherapie im Westen. In der ehemaligen Sowjetunion hingegen wurden Forschung, Produktion und Therapie mit Phagen fortgesetzt. Die Phagentherapie ist bis heute in einigen Nachfolgestaaten wie Georgien verbreitet [3]. Zunehmende Probleme mit MRE haben im Westen neues Interesse an der Phagentherapie geweckt, was dazu führte, dass in Belgien seit nahezu 15 Jahren Phagen in der Therapie komplexer Infektionen eingesetzt werden. Phagen werden mittlerweile in einigen weiteren europäischen Ländern (z. B. Frankreich) hergestellt und klinisch verwendet. Vor dem Hintergrund der aktuellen Evidenzlage zur Wirksamkeit der Phagentherapie und der Darstellung der klinischen Relevanz einzelner schwierig zu behandelnder Infektionen soll im vorliegenden Artikel die Bedeutung der Phagentherapie für Deutschland beleuchtet werden.

## Wirkungsweise der Phagentherapie

Die Phagentherapie basiert auf der gezielten Nutzung von Bakteriophagen (Viren von Bakterien), die hochspezifisch an ihre Wirtszellen binden, diese lysieren und damit eine bakterielle Infektion eliminieren können. Phagen vermehren sich am Infektionsort, solange dort geeignete Zielbakterien vorhanden sind, was eine selbstlimitierende Therapie ermöglicht. Sie stellen eine vielversprechende Alternative oder Ergänzung zu Antibiotika dar, insbesondere bei multiresistenten Bakterien [1, 4].

Ein entscheidender Aspekt der Phagentherapie ist die häufig stamm- und nicht speziesspezifische Wirksamkeit der Phagen. Im Gegensatz zu den oft breit wirksamen Antibiotika infizieren Phagen typischerweise nur spezifische Stämme innerhalb einer Bakterienspezies. Dies macht eine Identifikation wirksamer Phagen notwendig [1, 4, 5]. Diese hohe Spezifität hat sowohl Vorteile als auch Herausforderungen: Während sie das Mikrobiom des Patienten weitgehend schont und Nebenwirkungen minimiert, erfordert sie eine gezielte Auswahl passender Phagen für die jeweilige Infektion bzw. die Bestätigung der Wirksamkeit von Fertigpräparaten mit mehreren verschiedenen Phagen (Phagencocktails) vor der Therapie.

Zur Identifizierung wirksamer Phagen dient das sogenannte Phagogramm. Dieses überprüft, analog zum Antibiogramm für Antibiotika, welche spezifischen Phagen gegen den infizierenden bakteriellen Stamm wirksam sind, typischerweise basierend auf Plaque-Assays mit Agarose-Nährmediumplatten, die mit dem entsprechenden Bakterienisolat bewachsen sind [4, 5].

## Klinische Anwendung der Phagentherapie: Fertigpräparate versus individualisierte Anwendungen

Die Frage, ob Phagentherapeutika als Fertigpräparate (Phagencocktails „fertig aus dem Regal“) vermarktet oder personalisierte Ansätze verwendet werden, erfordert eine differenzierte Betrachtung. Aktuell dominieren fertige Cocktails die klinische Entwicklung. Dieser Ansatz eignet sich besonders für Erregerspezies mit breit wirksamen Phagen (*E. coli, P. aeruginosa, E. faecalis, E. faecium, S. aureus* und *S. epidermidis*). Dennoch gibt es im Vergleich zu herkömmlichen Pharmazeutika spezifische Besonderheiten zu berücksichtigen:

1. Phagencocktails können, wie die PhagoBurn-Studie zeigte, trotz stabiler Einzelphagen (mindestens 2‑jährige Haltbarkeit ohne Titerabfall) eine geringe Stabilität aufweisen (Abfall um 4–5 Größenordnungen in 6 Monaten; [6]). Deshalb sind Stabilitätsstudien essenziell, ebenso wie Maßnahmen wie stabilitätsfördernde Zusätze, Lagerung als Lyophilisat oder Abfüllung als Einzelphagen mit Kombination direkt vor der Behandlung.
2. Auch Fertigcocktails sollten idealerweise vor der Behandlung auf ihre Wirksamkeit gegen den spezifischen Patientenerreger getestet werden (Phagogramm).
3. Cocktails sollten zur Vermeidung der Resistenzbildung idealerweise verschiedene Phagen enthalten, sodass auch die unterschiedlichen Rezeptoren auf der Bakterienzelle erkannt werden können [7].
4. Einige Phagenpräparate müssen voraussichtlich an regionale zirkulierende Erregerstämme angepasst werden [8] und regelmäßig (etwa alle 2 Jahre) aktualisiert werden (persönliche Kommunikation mit Mzia Kutateladze, Eliava-Institut).

Für *K. pneumoniae* und *A. baumannii* ist die Entwicklung wirksamer Cocktails aufgrund der hohen Spezifität der Phagen und der großen Serotypenvariation deutlich schwieriger. Personalisierte Ansätze zur Phagentherapie könnten hier Erfolg versprechender sein. Dafür müssten Einzelphagen bereitgestellt werden, die z. B. auf Basis genomischer Surveillance der Serotypenverteilung ausgewählt werden [9].

Lösungsansätze für kommerzielle Phagencocktails könnten gentechnisch veränderte Phagen liefern, z. B. durch Erhöhung des Wirtsspektrums. Hierfür ist aber – im Vergleich mit natürlichen Phagen – noch viel Entwicklungsarbeit nötig. Für *Klebsiella*-Phagen wurde gezeigt, dass durch gentechnischen Austausch der Rezeptorbindungsdomänen neue Wirtsspezifitäten „transplantiert“ werden können [10]. Zudem existieren *Klebsiella*-Phagen mit mehreren solchen Domänen und somit breiterem Wirtsspektrum [11]. Kombiniert mit genetischen Surveillance-Daten könnten Phagen gezielt für spezifische epidemiologische Situationen designt werden.

## Wirksamkeit der Phagentherapie – Übersicht

Eine Zusammenfassung von 2001 aus Polen zeigte bei 1307 Patienten, die nicht auf ein verfügbares Antibiotikum ansprachen, in 1123 Fällen (85,9 %) eine vollständige Genesung mit Eradikation des bakteriellen Erregers und in 134 der Fälle (10,3 %) transiente klinische Verbesserungen (Erreger noch detektierbar; Tab. 1; [12]). Indikationen beinhalteten u. a. Mittelohrentzündungen, Meningitis, Pneumonien und Harnwegsinfektionen durch zahlreiche Erreger. Eine besonders gute Wirksamkeit der Phagentherapie (> 90 % Eradikation) zeigte sich bei eitriger Meningitis, eitriger Peritonitis, Furunkulose, Knocheninfektionen, chronisch eitrigen Fisteln und eitriger Mastitis.

**Tab. 1:** Ergebnisse der Phagentherapie nach Weber-Dabrowska et al. [12]. **Ec** *Escherichia coli*, **Kleb** *Klebsiella*, **Prot** *Proteus*, **Pseu** *Pseudomonas*, **Sa** *Staphylococcus aureus*

Klinische Diagnose	Erreger	Anzahl Patienten	Vollständige Genesung und Erregereradikation	Klinische Verbesserung, Erreger noch detektierbar	Kein Effekt
Sepsis	Sa, Ec, Kleb, Prot, Pseu	106	93 (87,7 %)	8 (7,5 %)	5 (4,7 %)
Eitrige Otitis media	Sa, Kleb, Pseu	33	28 (88,4 %)	3 (9,1 %)	2 (6,1 %)
Eitrige Meningitis	Sa, Ec, Kleb, Prot, Pseu	10	10 (100 %)	–	–
Variköse Geschwüre der unteren Extremitäten	Sa, Ec, Kleb, Prot, Pseu	77	47 (61 %)	21 (27,2 %)	9 (11,6 %)
Mukopurulente chronische Bronchitis, Laryngitis, Rhinitis	Sa, Ec, Kleb, Prot, Pseu	271	224 (82,6 %)	46 (16,9 %)	1 (0,3 %)
Bronchopneumonie, Empyem	Sa, Ec, Kleb, Prot, Pseu	57	47 (82 %)	10 (18 %)	–
Pleuritis mit Fistel	Sa, Ec, Kleb, Prot, Pseu	49	42 (86 %)	5 (10 %)	2 (4 %)
Eitrige Peritonitis	Sa, Ec, Kleb, Enterobacter, Prot, Pseu	66	60 (91 %)	5 (8 %)	1 (0,15 %)
Harnwegsinfektionen	Sa, Ec, Kleb, Prot, Pseu	78	59 (75,6 %)	9 (11,5 %)	10 (12,8 %)
Furunkulose	Sa	90	90 (100 %)	–	–
Dekubitus mit Infektion	Sa, Ec, Kleb, Prot, Pseu	16	13 (81 %)	–	3 (19 %)
Pyogene Arthritis und Myositis	Sa, Ec, Kleb, Prot, Pseu	19	17 (89 %)	–	2 (11 %)
Osteomyelitis der langen Knochen	Sa, Ec, Kleb, Prot, Pseu	40	38 (95 %)	–	2 (5 %)
Eitrige Osteitis nach Knochenfrakturen	Sa, Ec, Kleb, Prot, Pseu	41	37 (90 %)	–	4 (10 %)
Pyogene Infektionen von Verbrennungen	Sa, Ec, Kleb, Prot, Pseu	49	42 (86 %)	–	7 (14 %)
Pyogene postoperative Infektionen	Sa, Ec, Kleb, Pseu	35	29 (83 %)	–	6 (17 %)
Chronische eitrige Fisteln	Sa, Ec, Kleb, Prot, Pseu	180	168 (93 %)	–	12 (7 %)
Eitrige Sinusitis	Sa, Ec, Kleb, Prot, Pseu	46	38 (83 %)	3 (7 %)	5 (11 %)
Eitrige Mastitis	Sa, Ec	44	41 (93,1 %)	3 (6,8 %)	–
*Gesamt*	*–*	*1307*	*1123 (85,9* *%)*	*134 (10,3* *%)*	*50 (3,8* *%)*

Neuere Daten bestätigen die Wirksamkeit der Phagentherapie. Von 100 Behandlungen (2008–2022) v. a. in Belgien wurde in 77,2 % der Fälle eine klinische Verbesserung beobachtet, bei 61,3 % eine komplette Eradikation der Erreger [5]. Für die häufigsten Indikationen, Infektionen der unteren Atemwege (29 Fälle) und Knocheninfektionen (16 Fälle), waren die Phagenbehandlungen zu 62,1 % bzw. 81,3 % erfolgreich (Eradikation und/oder klinische Verbesserung). Einer israelischen Studie (2018–2022) zufolge waren Phagentherapien in 14/18 Fällen (77,8 %) mikrobiologisch erfolgreich (Reduktion oder Eradikation des Erregers; [13]). Von den 18 Fällen waren 13 durch Knochen‑, Gelenk- und Endoprotheseninfektionen bedingt. Hier wurde eine klinische Verbesserung und/oder Remission bei 11/13 Fällen (84,6 %) erreicht. Beide Studien beinhalten eine große Bandbreite von Indikationen und Erregerspezies.

Systematische Übersichtsarbeiten bestätigten diese Zahlen [14–16]. In 43 ausgewerteten Studien mit insgesamt 1469 Patienten führte die Therapie in 80,6 % der Fälle zur vollständigen Genesung und Erregereradikation und in weiteren 10,7 % zur klinischen Verbesserung mit noch detektierbarem Erreger (Tab. 2). Dies verdeutlicht das Potenzial der Phagentherapie zur Bekämpfung der zunehmenden Antibiotikaresistenz. Hier bestätigt sich auch die besonders gute Wirkung bei Knochen- und Gelenkinfektionen mit 93,1 % Erregereradikation und weiteren 3,3 % klinischer Verbesserung.

**Tab. 2:** Ergebnisse der Phagentherapie nach systematischen Übersichtsstudien

Klinische Diagnose	Erreger (Genus)	Anzahl Patienten	Vollständige Genesung und Erregereradikation	Klinische Verbesserung, Erreger noch detektierbar	Kein Effekt
Knochen- und Gelenkinfektionen [14]	*Acinetobacter, Enterococcus, Escherichia, Proteus, Pseudomonas, Staphylococcus*	277	258 (93,1 %)	9 (3,3 %)	10 (3,6 %)
Infektionen von Brandwunden [15]	*Escherichia, Enterococcus, Klebsiella, Pseudomonas, Proteus, Staphylococcus, Streptococcus*	111	55 (49,6 %)	31 (27,9 %)	25 (22,5 %)
Infektionen chronischer Wunden oder Ulcera [15]	*Escherichia, Enterococcus, Klebsiella, Pseudomonas, Proteus, Staphylococcus*	310	204 (65,8 %)	63 (20,3 %)	43 (13,9 %)
Dermatologische Infektionen [15]	*Escherichia, Pseudomonas, Staphylococcus, Streptococcus*	734	641 (87,3 %)	50 (6,8 %)	43 (5,9 %)
Infektionen in der Herz- und peripheren Gefäßchirurgie [16]	*Cutibacterium, Escherichia, Enterococcus, Proteus, Pseudomonas, Staphylococcus*	37	26 (70,3 %)	4 (10,8 %)	7 (18,9 %)
**Gesamt**	**–**	**1469**	**1184 (80,6** **%)**	**157 (10,7** **%)**	**128 (8,7** **%)**

Aus Sicht der evidenzbasierten Medizin (EbM) belegen die Daten aus den bisherigen Fallserien somit zumindest auf dem EbM-Level 4 (geringe Evidenz) das hohe Potenzial der Phagentherapie zur Bekämpfung der Antibiotikakrise. Zu betonen ist, dass keine einzige Studie der letzten Jahrzehnte klinisch relevante Nebeneffekte darstellen konnte, sodass von einer belegten Sicherheit und Verträglichkeit der Phagentherapie ausgegangen werden darf. Sie ist damit im Vergleich mit anderen antibakteriellen (Nichtantibiotika‑)Strategien wie der Inhibition mikrobieller regulatorischer Systeme durch Quorum-Sensing-Inhibitoren, den antimikrobiellen Peptiden und der fotodynamischen Therapie deutlich weiter fortgeschritten und ausgereifter [17].

## Potenzial der Phagentherapie für die wichtigsten Erreger

Die relevantesten Erregerspezies für Deutschland im Zusammenhang mit häufiger Multiantibiotikaresistenz sind *Escherichia coli, Klebsiella pneumoniae, Acinetobacter baumannii, Pseudomonas aeruginosa, Staphylococcus aureus, Staphylococcus epidermidis, Enterococcus faecium* und *Enterococcus faecalis* [18]. Der Bedarf an Phagentherapien für Deutschland (multiresistente und schwer behandelbare Infektionen) für diese Erreger liegt bei 62.000 pro Jahr. Im Folgenden wird für jeden dieser Erreger das Potenzial der Phagentherapie anhand klinischer Studien, Registerstudien und Einzelfallbeschreibungen abgeschätzt.

### Escherichia coli

*E. coli* verursacht u. a. Diarrhö, Harnwegsinfektionen, Sepsis und Pneumonien. Lytische, breit wirksame Phagen wurden beschrieben, was die Möglichkeit eines Phagencocktails als Fertigprodukt begünstigt. So zeigte der gentechnisch veränderte 4‑Phagen-Cocktail SNIPR001 (SNIPR Biome) Wirkung gegen > 90 % klinischer *E.-coli*-Isolate und in Mäusen eine selektive Entfernung von *E. coli* ohne Beeinträchtigung des Darmmikrobioms [19]. SNIPR Biome berichtete 2023 positive Ergebnisse ihrer Phase-1-Studie zu SNIPR001. Das Präparat soll bei Krebspatienten eine Sepsis durch translozierte Fluorchinolon-resistente *E. coli* verhindern. Die Studie bestätigte die Sicherheit und Verträglichkeit sowie eine Reduktion der *E.-coli*-Belastung im Darm [20].

Weitere aktuell laufende Studien:

- Eine Phase-1b-Studie (ClinicalTrials.gov ID NCT06559618) untersucht personalisierte Phagen gegen katheterassoziierte *E.-coli*-Harnwegsinfektionen.
- Intralytix testet in einer Phase-1/2a-Studie (ClinicalTrials.gov ID NCT03808103) einen Phagencocktail gegen adhärent invasive *E. coli* bei Morbus-Crohn-Patienten.
- Locus Biosciences untersucht einen 6‑Phagen-Cocktail (Clustered Regularly Interspaced Short Palindromic Repeats(CRISPR)-Cas-modifiziert) in einer Phase-2-Studie gegen unkomplizierte *E.-coli*-verursachte Harnwegsinfektionen. Erste Ergebnisse zeigen Sicherheit und schnelle, nachhaltige Bakterienreduktion mit Symptomfreiheit [21].

Einzelfallberichte beschreiben den Verlauf einer Harnwegsinfektion, bei der zwar keine Eradikation erreicht wurde, Mutationen des Erregers aber zu verringerter Virulenz und Symptomfreiheit führten [22], eine erfolgreich behandelte chronische Prostatitis mit vollständiger Eradikation und Symptomremission [23] und eine erfolgreich therapierte Urosepsis mit Erregereradikation [24].

### Klebsiella pneumoniae

*K. pneumoniae* verursacht u. a. Pneumonien, Harnwegsinfektionen und Sepsis [25], wobei zunehmende Multiresistenzen die Behandlung erschweren. Trotzt bekannter lytischer Phagen erschwert die hohe Kapselvielfalt (etwa 80 Typen) die Entwicklung breit wirksamer Cocktails [26]. *Klebsiella*-Phagen nutzen die bakterielle Kapsel oft als Rezeptor, wodurch ihre Wirksamkeit geografisch und zeitlich durch unterschiedliche Prävalenzen der Kapseltypen stark variieren kann. Evidenz zur Wirksamkeit Phagentherapie gegen *K. pneumoniae *stammt größtenteils aus Einzelfallberichten [27–40]:

- erfolgreiche Behandlungen von 2 Fällen chronischer Gelenkprotheseninfektionen [27, 28] und traumabedingten Knocheninfektionen [29, 37];
- Behandlungen von 5 Fällen chronischer Harnwegsinfektionen, davon 4 erfolgreich (Erregereradikation; [30, 32, 35, 36]) und einer nicht erfolgreich (behandelter Stamm zwar erfolgreich eliminiert, aber andere *K.-pneumoniae*-Stämme proliferierten nachfolgend; [39]);
- erfolgreiche Behandlungen eines Falls mit Kolonisation des Magen-Darm-Trakts, der Harnwege und eines Harnleiterstents [31] sowie eines Falls stentassoziierter Urosepsis [34];
- Behandlungen von 2 Fällen mit Lungeninfektionen, davon eine mit Eradikation [33] und eine mit Verbleib weniger virulenter *K.-pneumoniae*-Mutanten in der Lunge und verbessertem Gesundheitszustand [40];
- ein Fall eines Neugeborenen mit eitriger Meningitis, bei dem die Phagentherapie zur vollständigen Erregerfreiheit des Liquors führte [38].

### Pseudomonas aeruginosa

*P. aeruginosa* verursacht u. a. Wund‑, Harnwegs- und Lungeninfektionen (besonders bei Mukoviszidosepatienten), Otitis und Sepsis [41]. *P.-aeruginosa*-Phagen haben oft ein breites Wirkungsspektrum [42], was sie für Fertigpräparate attraktiv macht. Mehrere klinischen Studien untersuchten oder untersuchen phagentherapeutische Ansätze, darunter die abgeschlossene PhagoBurn-Studie (2015–2017), die erste klinische Untersuchung mit nach Good-Manufacturing-Practice-(GMP-)Standards hergestellten Phagen [6]. Der Phagencocktail reduzierte die Bakterienlast in Verbrennungswunden weniger effektiv als 1 %ige Sulfadiazin-Silber-Creme. Die Studie wurde vorzeitig abgebrochen, da die Phagenkonzentration durch starken Titerabfall des Phagencocktails unzureichend war.

Zwei Studien testen momentan vernebelt/inhalativ verabreichte Phagencocktails gegen *P. aeruginosa* bei Mukoviszidosepatienten. Das Präparat von BiomX zeigte in Phase 1b/2a gute Verträglichkeit, eine reduzierte Bakterienlast und verbesserte Lungenfunktion [43]. Der Cocktail der Phage4Cure-Studie (Charité Berlin) befindet sich in Phase 2 [4].

Eine Phase-1/2-Studie der Yale University zeigte zuvor die Sicherheit inhalierter Phagen [44], wurde jedoch wegen der COVID-19-Pandemie frühzeitig abgebrochen. Eine weitere Phase-1b/2a-Studie von Armata Pharmaceuticals (ClinicalTrials.gov ID NCT04596319) zu inhalativen Phagen bei Mukoviszidose- und Bronchiektasenpatienten (3- und 5‑Phagen-Cocktails) zeigte die Sicherheit und dosisabhängige Reduktion der Bakterienlast und führte zu einer mittlerweile abgeschlossenen Phase-2-Studie (ClinicalTrials.gov ID NCT05616221), bisher ohne veröffentlichte Ergebnisse. Ergebnisse einer weiteren Phase-1/2b-Studie mit einem intravenösen 4‑Phagen-Cocktail für Mukoviszidosepatienten (ClinicalTrials.gov ID NCT05453578) werden für Ende April 2025 erwartet. Eine Phase-1/2-Studie (ClinicalTrials.gov ID NCT06319235) zu einem Phagencocktail (Duofag) gegen *P. aeruginosa* und *S. aureus* für Operationsstelleninfektionen wird 2025 Ergebnisse liefern. TechnoPhage (Portugal) testete einen Phagencocktail gegen *P. aeruginosa, S. aureus* und *A. baumannii* bei diabetischen Fußgeschwüren in einer Phase-1/2a-Studie (ClinicalTrials.gov ID NCT048037089) erfolgreich (Sicherheit).

Registerstudien untermauern die Wirksamkeit der Phagentherapie. Von 100 Patienten in Pirnay et al. [5] wurden 38 wegen *P.-aeruginosa*-Infektionen (ohne Koinfektionen) behandelt. Dabei zeigten 30 (79 %) klinische Verbesserungen und 25 (66 %) eine vollständige Erregereradikation. Häufige Indikationen waren Lungeninfektionen (20 Fälle, davon 14 mit klinischer Verbesserung und 11 mit Eradikation) sowie Haut- und Weichteilinfektionen, z. T. mit Sepsis (8 Fälle, davon 7 mit Verbesserung und 6 mit Eradikation). Auch Patienten mit orthopädischen Protheseninfektionen (3/3), abdominellen Infektionen (2/2) und einer Hautinfektion an der Austrittsstelle eines Herzunterstützungsgeräts (LVAD-Driveline-Infektion; 1/1) wurden erfolgreich behandelt (klinische Verbesserung und Eradikation).

Im PhagoFlow-Projekt (2019–2024) kommen in Deutschland erstmals nach GMP-Richtlinien (Fraunhofer ITEM, Braunschweig) hergestellte Phagen und nach magistraler Rezeptur in einem personalisierten Therapieansatz zum klinischen Einsatz [4]. Erste Erfolge mit *P.-aeruginosa*-Phagen wurden z. B. bei chronischer Außenohrentzündung (topische Gabe) und chronischem Harnwegsinfekt erzielt. Im Rahmen des Projektes wurden die Phagen regelhaft in einem Phagogramm auf Wirksamkeit getestet.

Einzelfallstudien ergänzen das Bild. Rund 20 publizierte Fälle dokumentieren Behandlungserfolge, darunter eine Patientin mit refraktärer *P.-aeruginosa*-Harnwegsinfektion [45], die erfolgreich mit einem georgischen Phagenpräparat (klinische Heilung und Eradikation) behandelt wurde, sowie 3 erfolgreiche Sepsisbehandlungen [46–48].

### Acinetobacter baumannii

*A. baumannii* verursacht Infektionen wie beatmungsassoziierte Pneumonie, katheterassoziierte Blut- und Harnwegsinfektionen, Septikämie, Meningitis sowie Haut- und Weichteilinfektionen [49]. Wie bei *K. pneumoniae* ist der Hauptrezeptor für *A.-baumannii*-Phagen die bakterielle Kapsel, von der über 140 Typen bekannt sind [50]. Zur Abdeckung der meisten klinischen Stämme wären vermutlich über 300 Phagen erforderlich [1]. Bis auf die abgeschlossene Phase-1/2-Studie von TechnoPhage (Cocktail für diabetische Fußgeschwüre, s. oben) sind keine klinischen Studien registriert. Evidenz zur Wirksamkeit beruht v. a. auf Einzelfällen: auf einem Fall mit erfolgreicher Behandlung einer nekrotisierenden Pankreatitis [51], einem Fall traumabedingter Tibiainfektion [37] und 6 Fällen von Atemwegsinfektionen [52–54].

### Enterokokken

Enterokokken verursachen u. a. Harnwegsinfektionen, Sepsis, Endokarditis und Meningitis [55]. Enterokokken-Phagen können breit wirksam sein [56, 57]. Intralytix testet derzeit einen oralen Phagencocktail gegen Vancomycin-resistente Enterokokken im Gastrointestinaltrakt (Phase 1/2a, Abschluss 2025, ClinicalTrials.gov ID NCT05715619). Pirnay et al. [5] berichteten über eine erfolgreiche Behandlung einer chronischen Knocheninfektion mit *E. faecalis* durch einen intraläsional verabreichten Cocktail (Pyophage), einschließlich Eradikation. Vier weitere chronische Knocheninfektionen mit *E. faecalis* zusammen mit anderen Erregern zeigten klinische Verbesserung, bei 3/4 erfolgte eine vollständige Eradikation. Erfolgreich behandelt wurden auch Co-Infektionen mit *E. faecium*, darunter eine abdominelle Infektion und eine Sepsis, wobei Letztere zur vollständigen Eradikation führte. Rubalskii et al. [33] berichteten von einem Patienten mit einer Protheseninfektion durch *E. faecium, P. aeruginosa* und *S. aureus*, der durch lokal und oral verabreichte Phagen vollständig geheilt wurde. Drei weitere Fälle von *E.-faecalis*-Infektionen umfassen eine chronische Prostatitis (rektale oder kombinierte Gabe) sowie eine wiederkehrende Hüftprotheseninfektion (orale Gabe) mit klinischer Heilung und Eradikation [58–60].

### Staphylokokken

*S.-aureus*-Infektionen verursachen Sepsis, Endokarditis, Gelenk‑, Haut- und Weichteilinfektionen, pleuropulmonare und gerätebedingte Infektionen [61]. *S. epidermidis* besiedelt bevorzugt feuchte Hautstellen (Achselhöhlen, Leisten, Dammbereich) und vordere Nasenlöcher [62] und ist ein bedeutender nosokomialer Erreger bei implantatassoziierten Infektionen. Ein Fokus klinischer Entwicklungen liegt auf *S. aureus* wegen der klinischen Relevanz und der breiten Wirksamkeit von *S.-aureus*-Phagen, die sich für Fertigcocktails eignen [63]. Verschiedene Ansätze gegen *S. aureus* befinden sich in der klinischen Entwicklung; für diabetische Fußgeschwüre durch BiomX (positive Ergebnisse der Phase-2-Studie wurden im März 2025 berichtet; [43]) und TechnoPhage (Phase 1/2a abgeschlossen), für Operationsstelleninfektionen (die oben erwähnte Phase-1/2-Studie (Duofag-Cocktail) gegen *P. aeruginosa* und *S. aureus*) und für infizierte Hüft- und Kniegelenkprothesen durch Phaxiam (Phase-2-PhagoDAIR-Studie (ClinicalTrials.gov ID NCT05369104), Abschluss 2025, geplante größere Phase-2-GLORIA-Studie (ClinicalTrials.gov ID NCT06605651), 2025–2027). Eine abgeschlossene Phase-1-Studie zur persistierenden chronischen Rhinosinusitis testete einen Phagencocktail bei 9 *S.-aureus*-positiven Patienten (intranasal) und bestätigte die Verträglichkeit sowie die Erregereradikation bei 2/9 der Patienten [64]. Fedorov et al. [65] untersuchten Phagen-Antibiotika-Kombinationen bei Hüftgelenksinfektionen (*S. aureus* oder *S. epidermidis*). Nach Phagogramm-geführter Auswahl wurde ein Phagenpräparat (Microgen) in den Knochenzement integriert und postoperativ 10 Tage injiziert. Die Infektionsrate sank signifikant im Vergleich zur Kontrollgruppe (4,5 % vs. 36,4 % nach 12 Monaten).

Nach *P. aeruginosa *ist *S. aureus *der zweithäufigste Erreger in der Registerstudie von Pirnay et al. [5], mit 28/100 Fällen ausschließlich durch *S. aureus* verursacht. Von diesen wurden 24 (86 %) erfolgreich behandelt (klinische Verbesserung), mit Erregereradikation in 12 von 21 getesteten Fällen (57 %; 7 Fälle ohne abschließenden mikrobiologischen Test). Häufigste Indikationen waren Haut- und Weichteilinfektionen (klinische Verbesserung in 11/12 Fällen), Infektionen der oberen Atemwege (6/7 erfolgreich) und Knocheninfektionen (4/6 erfolgreich).

Einzelfallbeschreibungen sollen hier nicht vertieft werden; eine umfassende Übersicht bieten Plumet et al. [66]. In über 20 Fällen (Knochen‑, Gelenk‑, Haut‑, Weichteil‑, Lungen- und Herzinfektionen) wurden meist klinische Heilung und Erregereradikation berichtet. Phagen gegen *S. aureus* könnten zudem eine Therapieoption für atopisches Ekzem darstellen, da sie *S. aureus* ohne Schädigung des Hautmikrobioms gezielt entfernen können [67].

Das klinische Potenzial der Phagentherapie gegen *S. epidermidis* ist wenig erforscht. *S.-epidermidis*-Phagen sind häufig breit wirksam [68], einige sogar gegen *S. aureus, S. epidermidis* und andere Staphylokokken-Spezies [69]. Einzelfallberichte beschreiben u. a. eine erfolgreiche Behandlung einer chronischen Schultergelenkprotheseninfektion und 2 erfolgreich behandelte Co-Infektionen [5].

## Klinische Aktivitäten in der EU

Abschließend ist für alle genannten Erreger die Phagentherapie prinzipiell Erfolg versprechend. Auffallend ist, dass klinische Aktivitäten in der EU hauptsächlich in folgenden Ländern stattfinden:

- Frankreich: Firma Phaxiam (ehemals Pherecydes) mit GMP-Herstellungsfähigkeiten und den klinischen Studien PhagoBurn ([6]; abgeschlossen), PhagoDAIR (laufend) und GLORIA (geplant);
- Portugal: Fima TechnoPhage mit einer abgeschlossenen Phase-1/2-Studie für diabetische Fußgeschwüre und GMP-Herstellungsfähigkeiten;
- Tschechien: Firma MB Pharma mit laufender Phase-1/2-Studie des Duofag-Cocktails und GMP-Herstellungskapazitäten;
- Slowenien: Firma Jafral mit GMP-Herstellungsfähigkeiten.

Im Gegensatz dazu gibt es in Deutschland lediglich eine laufende klinische Studie (Phage4Cure) und keine kommerziellen GMP-Herstellungsfähigkeiten.

## Limitationen der Phagentherapie

Trotz des vielversprechenden Potenzials der Phagentherapie gibt es einige Limitationen, die eine breite klinische Anwendung aktuell erschweren.

Die derzeitige Evidenz zur Phagentherapie stammt überwiegend aus Fallserien und nichtrandomisierten Studien mit heterogenen Patientenkohorten und variierenden Endpunkten [5, 12–16]. Die meisten Arbeiten fallen in das EbM-Level 4, d. h., es fehlen robuste, randomisierte kontrollierte Studien (RCTs) zur verlässlichen Bestätigung der klinischen Wirksamkeit. Viele Fallberichte und Registerstudien weisen zudem keine standardisierte Definition für klinischen Erfolg oder bakterielle Eradikation auf, was die Vergleichbarkeit der Ergebnisse erschwert.

Eine zentrale Hürde für die routinemäßige Anwendung der Phagentherapie ist die Notwendigkeit eines Phagogramms, dessen Erstellung zeit- und ressourcenintensiv ist [4, 5]. Der gesamte Prozess, inkl. Vereinzelung des Erregers, dauert derzeit mehrere Tage und erfordert geschulte mikrobiologische Labore. Die damit verbundenen Verzögerungen sind insbesondere bei akuten Infektionen problematisch. Automatisierte Phagogramm-Technologien werden momentan entwickelt (z. B. von Vésale Bioscience, Belgien, und microphotonX, Deutschland), sind aber bislang nicht in der Klinik etabliert [4].

Eine weitere Herausforderung der Phagentherapie ist die mögliche Resistenzentwicklung von Bakterien gegen Phagen, z. B. durch Modifikation oder Verlust der Phagenrezeptoren an der Bakterienzelloberfläche, Abwehrsysteme wie CRISPR-Cas oder Biofilm-Bildung [70]. Die Resistenzentwicklung kann durch Phagencocktails, die unterschiedliche Phagen mit verschiedenen Rezeptorzielen kombinieren, oder durch gentechnisch modifizierte bzw. „trainierte“ [5] Phagen eingeschränkt werden.

Ein zusätzlicher limitierender Faktor sind polymikrobielle Infektionen. Während Antibiotika in der Regel ein breites Wirkspektrum aufweisen, sind Phagen spezifisch für einzelne bakterielle Stämme. Dies bedeutet, dass bei Mischinfektionen mehrere Phagen parallel eingesetzt werden müssen, was die Komplexität der Therapie erhöht [37]. Aktuell gibt es nur wenige Studien, die sich mit der Optimierung von Phagenkombinationen für polymikrobielle Infektionen befassen [71].

Die Herstellung von Phagen unter GMP-Bedingungen stellt eine weitere Herausforderung dar. Derzeit erfolgt die Produktion durch Kultivierung von Wirtsbakterien, die mit Phagen infiziert werden. Dieser Prozess ist aufwendig, da geeignete Wirtsbakterien in einer Master Cell Bank (MCB) vorgehalten werden müssen und Endotoxin-Verunreinigungen entfernt werden müssen, um eine sichere therapeutische Anwendung zu gewährleisten. Eine vielversprechende Alternative ist die zellfreie Herstellung von Phagen, die von Invitris (München) entwickelt wird [72]. Dieser Ansatz würde die Produktion ohne MCB und Endotoxin-Entfernung ermöglichen, was die Kosten senken und die Phagentherapie zugänglicher machen könnte.

## Fazit

Die Phagentherapie ist vielversprechend im Kampf gegen antibiotikaresistente Infektionen und könnte das Gesundheitssystem erheblich entlasten. Trotz laufender klinischer Studien und einer durchschnittlichen Wirksamkeit von etwa 80 % bleibt die Anwendung in Deutschland mit heute weniger als 100 Behandlungen pro Jahr deutlich hinter den Möglichkeiten zurück. Durch gezielte Maßnahmen könnte bereits jetzt die Versorgungslage deutlich verbessert werden, um schon vor Verfügbarkeit kommerzieller Phagenpräparate Patienten behandeln zu können und die Zukunftsfähigkeit Deutschlands bzgl. der Phagentherapie zu sichern.

1. Kommerzielle Hersteller mit GMP-Zertifizierung existieren im EU-Ausland, aber nicht in Deutschland. Die Einrichtung eines deutschen Phagenherstellungsbetriebs würde Kliniken den Zugang zu Phagen erleichtern und Abhängigkeiten von ausländischen Herstellern reduzieren. So könnten Patienten mit nicht behandelbaren schweren Infektionen in Deutschland bereits heute vor einer zukünftigen Zulassung von fertigen Phagentherapeutika (Cocktails) im individualisierten Ansatz, den die magistrale Rezeptur ermöglicht, behandelt werden.
2. Auch regulatorisch könnte der Zugang zu Phagen vereinfacht werden. Belgien nimmt in Westeuropa eine Vorreiterrolle ein, indem es Behandlungen mit Phagenwirkstoffen ohne GMP-Zertifikat erlaubt (Qualitätsanforderungen sind in einer Monografie festgelegt; [73]). Portugal führte Ende 2024 nach einer Petition von Ärzten und Patienten ein ähnliches Konzept ein. Eine solche Regelung für Deutschland würde patientenspezifische Anwendungen außerhalb strikter GMP-Vorgaben ermöglichen.
3. Die Entwicklung innovativer Technologien wie der zellfreien Herstellung oder automatisierter Diagnostik muss vorangetrieben werden, um Produktionskosten zu senken und die Logistik zu vereinfachen.
4. Die gezielte Förderung kontrollierter, randomisierter klinischer Studien ist entscheidend, um die Entwicklung und Verbreitung der Phagentherapie zu beschleunigen. Darüber hinaus sollten sämtliche Einzelfallbehandlungen systematisch in einem Patientenregister erfasst werden. Dieses Register könnte nicht nur als ergänzender Wirksamkeitsnachweis dienen – auch im Kontext von Zulassungsverfahren –, sondern auch wertvolle Erkenntnisse für das Design zukünftiger klinischer Studien liefern, z. B. in Bezug auf besonders geeignete Indikationen, Phagendosierungen oder besonders wirkungsvolle Kombinationen mit Antibiotika.
